# Suicide Ideation of Individuals in Online Social Networks

**DOI:** 10.1371/journal.pone.0062262

**Published:** 2013-04-26

**Authors:** Naoki Masuda, Issei Kurahashi, Hiroko Onari

**Affiliations:** 1 Department of Mathematical Informatics, The University of Tokyo, 7-3-1 Hongo, Bunkyo, Tokyo, Japan; 2 iAnalysis LLC, 2-2-15 Minamiaoyama, Minato-ku, Tokyo, Japan; Hungarian Academy of Sciences, Hungary

## Abstract

Suicide explains the largest number of death tolls among Japanese adolescents in their twenties and thirties. Suicide is also a major cause of death for adolescents in many other countries. Although social isolation has been implicated to influence the tendency to suicidal behavior, the impact of social isolation on suicide in the context of explicit social networks of individuals is scarcely explored. To address this question, we examined a large data set obtained from a social networking service dominant in Japan. The social network is composed of a set of friendship ties between pairs of users created by mutual endorsement. We carried out the logistic regression to identify users’ characteristics, both related and unrelated to social networks, which contribute to suicide ideation. We defined suicide ideation of a user as the membership to at least one active user-defined community related to suicide. We found that the number of communities to which a user belongs to, the intransitivity (i.e., paucity of triangles including the user), and the fraction of suicidal neighbors in the social network, contributed the most to suicide ideation in this order. Other characteristics including the age and gender contributed little to suicide ideation. We also found qualitatively the same results for depressive symptoms.

## Introduction

Suicide is a major cause of death in many countries. Japan possesses the highest suicide rate among the OECD countries in 2009 [1]. In fact, suicide explains the largest number of death cases for Japanese adolescents in their twenties and thirties [1]. Suicide is also a major cause of death for youths in other countries including the United States [2].

Since the seminal sociological study by Durkheim in the late nineteenth century [3], suicides have been studied for both sociology interests and public health reasons. In particular, Durkheim and later scholars pointed out that social isolation, also referred to as the lack of social integration, is a significant contributor to suicidal behavior [3]–[6]. Roles of social isolation in inducing other physical and mental illnesses have also been examined [7]. Conceptual models that inherit Durkheim’s idea also claim that social networks affect general health conditions including tendency to suicide [8]–[11].

Social network analysis provides a pragmatic method to quantify social isolation [12], [13]. In their seminal work, Bearman and Moody explicitly studied the relationship between suicidal behavior and egocentric social networks for American adolescents using data obtained from a national survey (National Longitudinal Study of Adolescent Health) [14]. They showed that, among many independent variables including those unrelated to social networks, a small number of friends and a small fraction of triangles to which an individual belongs significantly contribute to suicide ideation and attempts. A small number of friends is an intuitive indicator of social isolation. Another study derived from self reports from Chinese adolescents also supports this idea in a quantitative manner [15]. The paucity of triangles, or intransitivity [12], also characterizes social isolation [14]. Individuals without triangles are considered to lack membership to social groups even if they have many friends [16]; social groups are often approximated by overlapping triangles [17], [18].

Nevertheless, the structure of the Bearman–Moody study [14] implies that our understanding of relationships between social networks and suicide is still limited. First, in the survey, a respondent was allowed to list best five friends of each gender. However, many respondents would generally have more friends. The imposed upper limit may distort network-related personal quantities such as the number of friends and triangles. Second, their study was confined inside each school in the sense that only in-school names are matched. If a respondent X named two out-school friends that were actually friends of each other, the triangle composed of these three individuals was dismissed from the analysis. Therefore, the accuracy of the triangle counts in their study may be limited such that the relationship between intransitivity and suicidal behavior remains elusive.

In the present study, we examine the relationship between social networks and suicide ideation using a data set obtained from a dominant social networking service (SNS) in Japan, named mixi. Our approach addresses limitations in the previous study [14]. First, an entire social network of users is available, where a link between two users represents explicit bidirectional friendship endorsed by both users. Some users have quite a large number of friends, as in general social networks [13]. Second, for the same reason, we can accurately calculate the number of triangles for each user. An additional feature of the present data set is that the sample is relatively diverse because anybody can register for free. In contrast, the respondents were 7 to 12 graders in schools in the Bearman–Moody study.

A function of mixi relevant to this study is user-defined communities. A community is a group of users that get together under a common interest, such as hobby, affiliation, or creed. A user-defined community of mixi is often composed of users that have not known each other beforehand. Although some SNSs have user-defined communities, and their dynamics were studied [19], major SNSs including Facebook do not own this type of user-defined communities. We define suicide ideation by the membership of a user to at least one community related to suicide. Then, we statistically compare users with and without suicide ideation in terms of users’ properties including those related to egocentric networks.

## Results

### Multivariate Logistic Regression

We defined the group of users with suicide ideation and the control group of users, as described in Methods. Table 1 indicates that the difference in the mean of each independent variable (see Methods for the definition of the independent variables) between the suicide and control groups is significant (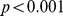, Student’s 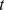-test). We also verified that the distributions of each independent variable are also significantly different between the two groups (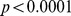, Kolmogorov-Smirnov test).

**Table 1:** Univariate statistics of independent variables for the suicide and control groups.

	Suicide group		Control group		
Variable	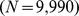		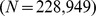		
	Mean 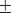 SD	Range	Mean 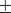 SD	Range	*p*-value
		(min,max)		(min,max)	
Age	27.4 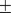 10.3	(17, 97)	27.7 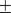 9.2	(14, 96)	0.000652
Community number	283.7 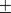 284.3	(1, 1000)	46.3 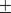 79.4	(1, 1000)	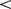 0.0001
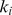	82.9 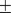 98.7	(2, 1000)	65.8 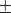 67.6	(2, 1000)	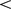 0.0001
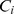	0.087 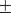 0.097	(0, 1)	0.150 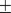 0.138	(0, 1)	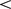 0.0001
Homophily (suicide)	0.0110 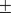 0.0329	(0, 1.000)	0.0012 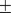 0.0080	(0, 0.667)	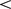 0.0001
Registration period	1235.7 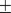 638.9	(122, 2878)	1333.5 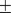 670.5	(102, 2891)	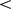 0.0001
Gender (female)	5,786 (57.9 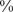 )		126,941 (55.4 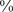 )		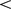 0.0001
No. suicidal communities	1.20 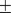 0.51	(1, 4)	N/A	N/A	N/A
No. login days	28.9 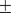 4.4	(1, 31)	26.9 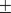 6.3	(1, 31)	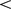 0.0001

The *p*-value for the gender is based on the Chi-square test. The *p*-values for the other independent variables are based on the Student’s *t*-test. Also shown are the statistics of two auxiliary variables that are not used in the logistic regression, i.e., the number of suicidal communities to which the user belongs and the number of days on which the user logged on to mixi. The *p*-value for the number of log-on days is based on the Student’s *t*-test. SD: standard deviation.

The results obtained from the multivariate logistic regression are summarized in Table 2. The VIF values (see Methods) are much less than 5 for all the independent variables. The three types of correlation coefficients between pairs of the independent variables are also sufficiently small (Table 3). On these bases, we justify the application of the multivariate logistic regression to our data.

**Table 2:** Multivariate logistic regression of suicide ideation on individual and network variables.

Variable	OR	CI	*p*-value	VIF
Age	1.00463	(1.00211, 1.00716)	0.000313	1.091
Gender (female = 1)	0.821	(0.783, 0.861)	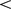 0.0001	1.028
Community number	1.00733	(1.00720, 1.00747)	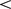 0.0001	1.197
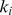	0.99790	(0.99758, 0.99821)	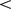 0.0001	1.156
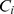	0.0093	(0.0069, 0.0126)	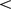 0.0001	1.081
Homophily (suicide)	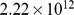	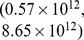	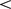 0.0001	1.016
Registration period	0.999383	(0.999346, 0.999420)	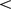 0.0001	1.135

OR: odds ratio; CI: 95% confidence interval; VIF: variance inflation factor.

**Table 3:** Correlation coefficients between pairs of independent variables for the suicide, depression, and control groups.

Variable 1	Variable 2	Suicide			Depression			Control		
		P	S	K	P	S	K	P	S	K
Age	Gender	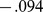	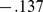	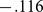	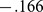	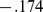	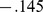	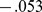	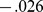	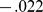
Age	Community number	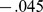	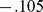	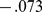	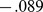	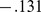	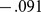	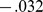	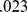	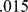
Age	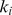	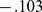	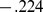	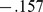	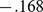	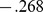	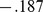	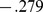	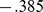	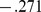
Age	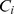	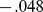	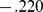	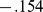	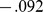	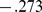	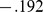	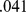	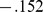	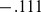
Age	Homophily (suicide)	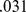	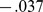	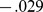	N/A	N/A	N/A	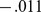	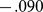	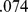
Age	Homophily (depression)	N/A	N/A	N/A	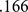	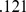	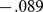	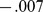	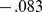	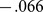
Age	Registration period	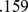	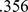	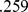	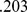	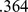	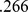	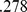	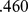	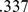
Gender	Community number	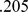	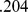	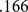	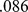	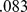	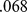	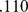	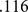	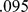
Gender	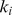	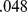	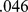	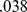	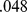	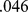	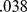	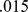	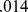	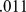
Gender	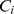	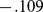	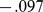	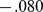	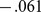	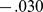	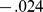	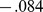	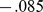	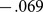
Gender	Homophily (suicide)	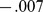	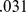	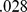	N/A	N/A	N/A	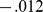	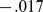	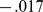
Gender	Homophily (depression)	N/A	N/A	N/A	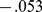	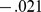	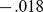	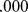	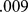	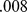
Gender	Registration period	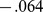	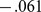	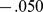	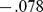	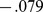	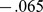	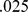	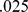	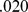
Community number	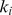	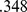	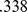	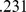	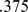	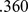	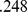	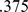	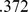	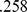
Community number	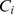	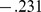	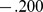	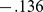	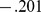	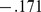	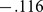	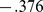	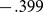	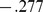
Community number	Homophily (suicide)	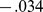	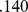	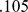	N/A	N/A	N/A	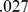	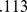	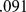
Community number	Homophily (depression)	N/A	N/A	N/A	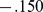	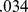	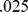	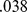	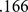	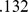
Community number	Registration period	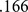	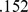	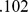	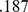	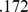	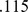	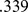	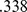	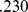
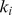	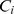	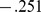	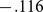	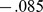	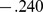	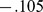	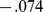	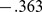	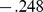	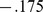
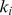	Homophily (suicide)	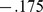	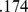	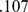	N/A	N/A	N/A	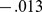	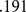	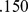
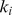	Homophily (depression)	N/A	N/A	N/A	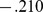	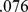	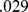	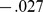	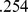	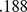
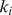	Registration period	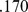	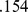	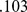	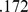	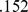	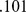	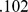	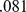	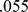
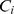	Homophily (suicide)	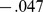	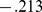	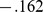	N/A	N/A	N/A	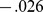	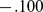	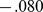
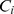	Homophily (depression)	N/A	N/A	N/A	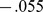	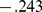	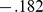	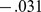	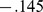	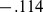
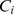	Registration period	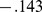	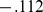	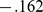	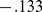	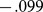	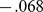	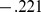	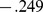	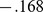
Homophily (suicide)	Registration period	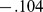	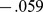	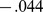	N/A	N/A	N/A	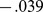	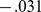	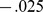
Homophily (depression)	Registration period	N/A	N/A	N/A	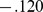	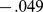	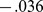	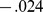	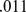	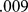

P: Pearson; S: Spearman; K: Kendall correlation coefficients.

The odds ratio (OR) values shown in Table 2 suggest the following. A one-year older user is 1.00463 times more likely to belong to the suicide group than the control group on average. Likewise, being female, membership to one community, having one friend, an increase in 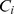 by 0.01, an increase in the fraction of friends in the suicide group (i.e., homophily variable) by 0.01, and one day of the registration period make a user 0.821, 1.00733, 0.99790, 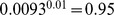, 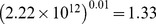, and 0.999383 times more likely to belong to the suicide group, respectively. For all the independent variables, the 95% confidence intervals of the ORs do not contain unity, and the 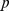-values are small. Therefore, all the independent variables significantly contribute to the regression. In addition, because the AUC (see Methods) is large (i.e. 0.873), the estimated multivariate logistic model captures much of the variation in the user’s behavior, i.e., whether to belong to the suicide group or not.

### Univariate Logistic Regression

All the independent variables significantly contribute to the multivariate regression probably because of the large sample size of our data set. Therefore, we carried out the univariate logistic regression between the dependent variable (i.e., membership to the suicide versus control group) and each independent variable to better clarify the contribution of each independent variable.

The results obtained from the univariate logistic regression are shown in Table 4. Although the 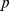-value for each independent variable is small, the AUC value considerably varies between different independent variables. The ORs for the community number, local clustering coefficient, homophily, and registration period are consistent between the multivariate and univariate regressions. For example, both regressions indicate that a user with a large community number tends to belong to the suicide group. These independent variables also yield large AUC values under the univariate regression.

**Table 4:** Univariate logistic regression of suicide ideation on individual and network variables.

Variable	OR	CI	*p*-value	AUC
Age	0.99604	(0.99377, 0.99832)	0.000651	0.515
Gender (female = 1)	1.106	(1.062, 1.152)	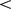 0.0001	0.512
Community number	1.00728	(1.00716, 1.00741)	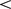 0.0001	0.867
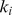	1.00259	(1.00237, 1.00280)	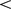 0.0001	0.549
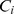	0.000581	(0.000428, 0.000789)	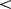 0.0001	0.690
Homophily (suicide)	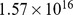	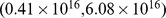	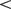 0.0001	0.643
Registration period	0.999783	(0.999753, 0.999813)	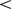 0.0001	0.545

OR: odds ratio; CI: 95% confidence interval; AUC: area under the curve.

The community number makes by far the largest contribution among the seven independent variables. The AUC value obtained from the univariate regression (0.867) is close to that obtained by the multivariate regression (0.873).

The independent variable with the second largest explanatory power is the local clustering coefficient (AUC 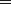 0.690). The results are consistent with the previous ones [14]. We stress that we reach this conclusion using a data set whose full social network is available.

The homophily variable makes the third largest contribution (AUC 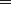 0.643). Although we refer to this independent variable as homophily (see Methods), the effect of this variable is in fact interpreted as either homophily or contagion [20], [21]. Nevertheless, the result is consistent with previous claims that suicide is contagious (for recent accounts, see [6], [22]–[26]; but see [27] for a critical review) and that other related states such as depressive symptoms are contagious [28], [29] (but see [30], [31]).

The effect of the age, gender, and degree (i.e., number of friends), on suicide ideation is small, yielding small AUC values, close to the minimum value 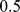 (Table 4). In addition, the ORs for these variables are inconsistent between the multivariate and univariate regressions. For example, a female user is more likely to belong to the suicide group according to the univariate regression and vice versa according to the multivariate regression. Therefore, we conclude that these three independent variables do not explain suicide ideation.

The registration period also yields a small AUC value (i.e., 0.545). Therefore, suicide ideation depends on the community number, local clustering coefficient, and homophily variable not because they commonly depend on the registration period.

### Depressive Symptoms

Our data set allows us to investigate correlates between users’ other characteristics and the independent variables if the characteristics have corresponding used-defined communities in the SNS. We repeated the same series of analysis for depressive symptoms, which are suggested to be implicated in suicidal behavior [5], [22], [32]. A user is defined to own depressive symptoms when the user belongs to at least one of the seven depression-related communities (Methods).

**Figure 1:**
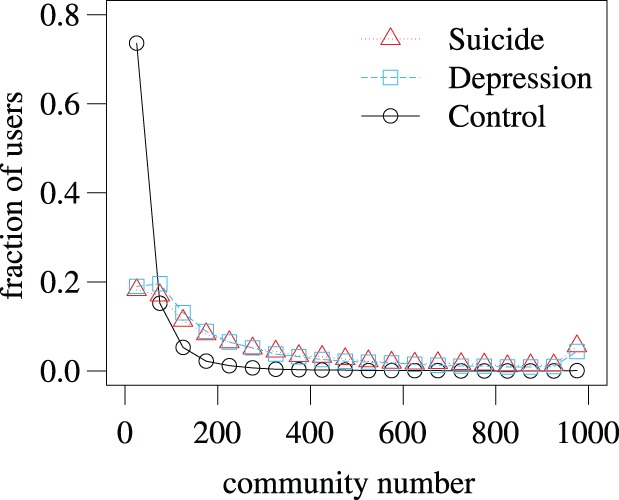
Distribution of the community number (i.e., number of communities to which a user belongs) for the suicide, depression, and control groups. We set the bin width for generating the histogram to 50. The abrupt increase in the distribution at 1000 communities for the suicide and depression groups is owing to the restriction that a user can belong to at most 1000 communities.

**Figure 2:**
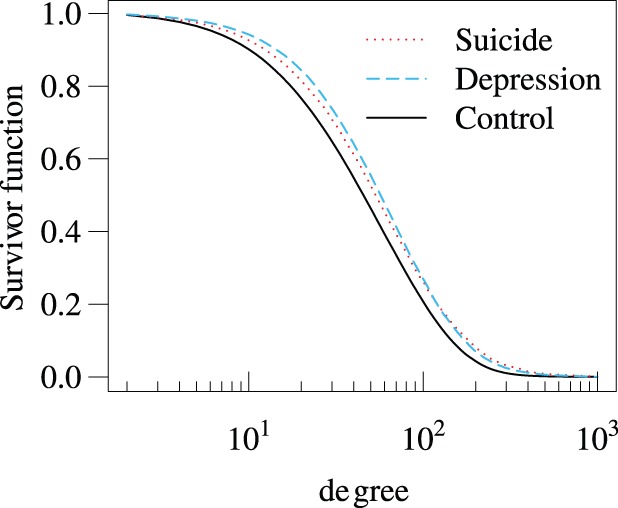
Complementary cumulative distribution of the degree (i.e., fraction of users having the degree larger than a specified value) for the suicide, depression, and control groups.

**Figure 3:**
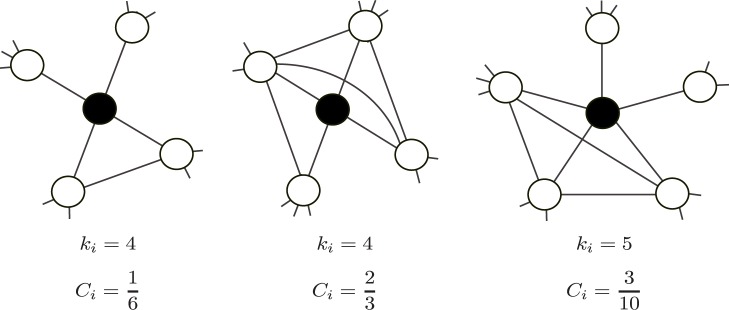
Dependence of the mean local clustering coefficient on the degree for the suicide, depression, and control groups. Each data point 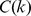 for degree 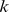 is obtained by averaging 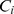 over the users in a group with degree 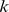. Large fluctuations of 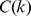 at large 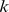 values are caused by the paucity of users having large 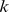.

The statistics of the independent variables for the depression group are compared with those for the control group in Figures 1, 2, 3, and Table 5. Each independent variable in the depression and control groups is significantly different in terms of the mean (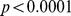, Student’s 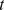-test; see Table 5) and distribution (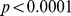, Kolmogorov-Smirnov test).

**Table 5:** Univariate statistics of independent variables for the depression and control groups.

	Depression group		Control group		
Variable	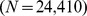		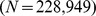		*p*-value
	Mean 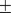 SD	Range	Mean 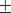 SD	Range	
		(min,max)		(min,max)	
Age	28.8 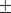 9.4	(16, 97)	27.7 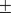 9.2	(14, 96)	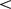 0.0001
Community number	249.6 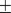 263.1	(1, 1000)	46.3 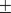 79.4	(1, 1000)	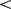 0.0001
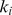	81.9 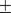 88.1	(2, 1000)	65.8 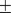 67.6	(2, 1000)	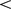 0.0001
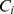	0.085 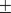 0.089	(0, 1)	0.150 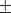 0.138	(0, 1)	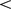 0.0001
Homophily (depression)	0.0196 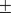 0.0501	(0, 1.000)	0.0031 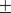 0.0131	(0, 0.667)	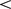 0.0001
Registration period	1389.4 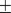 659.2	(122, 2885)	1333.5 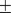 670.5	(102, 2891)	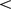 0.0001
Gender (female)	16,872 (69.1 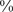 )		126,941 (55.4 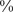 )		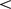 0.0001
No. suicidal communities	1.16 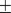 0.47	(1, 6)	N/A	N/A	N/A
No. login days	28.8 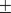 4.4	(1, 31)	26.9 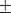 6.3	(1, 31)	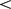 0.0001

The values for the control group are equal to those shown in Table 1 except for those of the homophily variable. The homophily is defined as the fraction of neighbors belonging to the depression group in this table, whereas it is defined as the fraction of neighbors belonging to the suicide group in Table 1. The *p*-value for the gender is based on the Chi-square test. The *p*-values for the other variables are based on the Student’s *t*-test. SD: standard deviation.

We applied the multivariate and univariate logistic regressions to identify independent variables that contribute to depressive symptoms (i.e., membership to the depression group). The control group is the same as that used for the analysis of suicide ideation. The results are shown in Tables 6 and 7. The VIF values shown in Table 6 and the correlation coefficient values shown in Table 3 qualify the use of the multiple logistic regression. The results are qualitatively the same as those for the suicide case.

**Table 6:** Multivariate logistic regression of depressive symptoms on individual and network variables.

Variable	OR	CI	*p*-value	VIF
Age	1.0141	(1.0124, 1.0158)	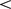 0.0001	1.104
Gender (female = 1)	1.532	(1.481, 1.585)	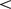 0.0001	1.019
Community number	1.00790	(1.00778, 1.00803)	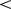 0.0001	1.155
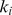	0.99833	(0.99810, 0.99856)	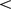 0.0001	1.154
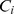	0.0145	(0.0118, 0.0178)	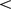 0.0001	1.079
Homophily (depression)	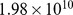	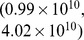	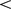 0.0001	1.022
Registration period	0.999744	(0.999720, 0.999769)	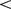 0.0001	1.117

OR: odds ratio; CI: 95% confidence interval; VIF: variance inflation factor.

**Table 7:** Univariate logistic regression of depressive symptoms on individual and network variables.

Variable	OR	CI	*p*-value	AUC
Age	1.0110	(1.0097, 1.0123)	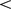 0.0001	0.551
Gender (female = 1)	1.799	(1.748, 1.850)	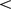 0.0001	0.568
Community number	1.00826	(1.00814, 1.00837)	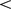 0.0001	0.860
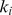	1.00258	(1.00243, 1.00274)	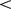 0.0001	0.566
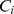	0.000415	(0.000338, 0.000509)	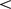 0.0001	0.692
Homophily (depression)	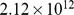	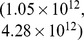	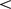 0.0001	0.658
Registration period	1.000126	(1.000106, 1.000145)	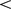 0.0001	0.522

OR: odds ratio; CI: 95% confidence interval; AUC: area under the curve.

## Discussion

We investigated relationships between suicide ideation and personal characteristics including social network variables using the data obtained from a major SNS in Japan. We found that an increase in the community number (i.e., the number of user-defined communities to which a user belongs), decrease in the local clustering coefficient (i.e., local density of triangles, or transitivity), and increase in the homophily variable (i.e., fraction of neighboring users with suicide ideation) contribute to suicide ideation by the largest amounts in this order. In addition, the results are qualitatively the same when we replaced suicide ideation by depressive symptoms. Remarkably, the most significant three variables represent online social behavior of users rather than demographic properties such as the age and gender.

Our result that the age and gender little influence suicide ideation is inconsistent with previous findings [6]. The weak age effect in our result may be because the majority of registered users is young; the mean age of the users in the control group is 27.7 years old (Table 1). Nevertheless, we stress that suicide is a problem particularly among young generations to which a majority of the users belong.

We concluded that the node degree little explains suicide ideation. In contrast, previous studies showed that suicidal behavior is less observed for individuals with more friends [14], [15]. It has also been a long-standing claim that social isolation elicits suicidal behavior [3]–[6]. As compared to typical users, some users may spend a lot of time online to gain many ties with other users and belong to many communities on the SNS. Such a user may be active exclusively online and feel lonely, for example, to be prone to suicide ideation. Although this is a mere conjecture, such a mechanism would also explain the strong contribution of the community number to suicide ideation revealed in our analysis. In contrast, many people nowadays, especially the young, regularly devote much time to online activities including SNSs [33]. Therefore, the data obtained from SNSs may capture a significant part of users’ real lives.

Because mixi enjoys a large number of users and implements the user-defined community as a main function, its user-defined communities cover virtually all major topics. Therefore, applying the present methods to other psychiatric illness and symptoms, such as schizophrenia, bipolar disorder, and alcohol abuse, as well as positive symptoms may be profitable.

Our studies are limited in some aspects. First, we identified suicide ideation with the membership to a relevant community, but not with suicide attempts or committed suicides. Second, membershipship to a relevant community may not even imply suicide ideation. Users may enter the suicide group because they have encountered suicide among their friends or family. Third, our data are a specific sample of individuals from a general population. This criticism applies to any work that relies on SNS data. However, it is particularly pertinent when one focuses on individuals’ chracteristics (e.g., personality and attitudes) rather than collective phenomena online (e.g., contagion on SNSs). Although it is beyond the scope of the current study, quantifying the extent to which our sample accurately represents general populations remains a future challenge.

## Methods

### Data

Mixi is a major SNS in Japan. It started to operate on March 2004 and enjoys more than 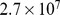 registered users as of March 2012. Similar to other known SNSs, users of mixi can participate in various activities such as making friendship with other users, writing microblogs, sending instant messages to others, uploading photos, and playing online games. Registration is free. See [34] for a previous study of the mixi social network.

In mixi, there were more than 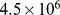 user-defined communities on various topics as of April 2012. Users can join a user-defined community if the owner personally permits or the owner allows anybody to join it.

We identified suicide ideation with the membership of a user to at least one suicidal community. To define suicidal community, which is sufficiently active, we first selected communities satisfying the following five criteria: (1) The name included the word “suicide” (“jisatsu” in Japanese), (2) there were at least 1000 members on November 2, 2011, (3) there were at least 100 comments posted on October, 2011, which were directed to other comments or topics, (4) there were at least three independent topics on which comments were made on October, 2011, and (5) the condition for admission was made open to public. Seven communities met these criteria. Then, we excluded one community whose name indicated that it concentrated on methodologies of committing suicide and two communities whose names indicated that they encouraged members to live with hopes (one contained the word “want to live”, and the other contained the word “have a fun” in their names; translations by the authors).

As a result, four communities were qualified as suicidal communities. The user statistics of these communities are shown in Table 8. A user that belongs to at least one suicidal community is defined to possess suicide ideation. To exclude inactive users, we restricted ourselves to the set of active users. The active user was defined as users that existed as of January 23, 2012 and logged on to mixi in more than 20 days per month on average from August through December 2011. A similar definition was used in a previous study of the Facebook social network [35]. We also discarded users with zero or one friend on mixi because the triangle count described below was undefined for such users. Despite this exclusion, the remaining data allowed us to examine the effect of social isolation in terms of the degree, i.e., number of neighbors, because the degree was widely distributed between 2 and 1000. There were 9990 active users with suicide ideation (suicide group).

**Table 8:** Statistics of suicidal communities.

ID	Date of creation	No. users	No. active	Fraction of	No.	No. active
	(day/month/year)		users	active users ( 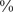 )	comments	topics
1	18/01/2008	8367	5985	69.9	741	16
2	21/09/2006	5135	3192	62.9	318	6
3	01/12/2004	3459	1883	53.2	279	12
4	04/02/2008	1445	965	62.4	105	9

We statistically compared the users in the suicide group with users without suicide ideation. Because the number of users was huge, we randomly selected 228949 active users that possessed at least two friends and belonged to neither of the seven candidates of the suicidal community defined above nor the ten candidates of the depression-related community defined below. We call this set of users the control group.

The employees of mixi deleted private information irrelevant to the present study and encrypted the relevant private information before we analyzed the data. In addition, we conducted all the analysis in the central office of mixi located in Tokyo using a computer that was not connected to Internet.

### Statistical Models

The dependent variable that represents the level of suicide ideation is binary, i.e., whether a user belongs to a suicidal community or not. Therefore, we used univariate and multivariate logistic regressions. To check the multicollinearity between independent variables to justify the use of the multivariate logistic regression, we carried out two subsidiary analysis. First, we measured the variance inflation factor (VIF) for each independent variable (see [36], [37] and references therein). The VIF is the reciprocal of the fraction of the variance of the independent variable that is not explained by linear combinations of the other independent variables. It is recommended that the VIF value for each independent variable is smaller than 10 (preferably smaller than 5) for the multivariate logistic regression to be valid. Second, we measured the Pearson, Spearman, and Kendall correlation coefficients between the independent variables.

To quantify the explanatory power of the logistic model, we measured the area under the receiver operating characteristic curve (AUC) for each fit (e.g., [37]). The receiver operating characteristic curve is the trajectory of the false positive (i.e., fraction of users in the control group that are mistakenly classified into the suicide group on the basis of the linear combination of the independent variables) and the true positive (i.e., fraction of users in the suicide group correctly classified into the suicide group), when the threshold for classification is varied. The AUC value falls between 0.5 and 1. A large AUC value indicates that the logistic regression fits well to the data in the sense that users are accurately classified into suicide and control groups.

### Independent Variables

We considered seven independent variables. Their univariate statistics for the suicide and control groups are shown in Table 1.

#### Demographics

Demographic independent variables include age and gender. Our analysis does not include ethnic components because most users are Japanese-speaking Japanese; mixi provides services in Japanese. Other demographic, socioeconomic, and personal characteristic variables such as residence area, occupation, company/school, and hobby, were not used because they were unreliable. In fact, many users leave them blank or do not fill them consistently, probably because they do not want to disclose them.

#### Community number

The number of user-defined communities that a user belongs to was adopted as an independent variable. We refer to this quantity as community number. The community number obeys a long tailed distribution for both suicide and control groups (Figure 1). The mean is quite different between the two groups (Table 1).

#### Degree

When a user sends a request to another user and the recipient accepts the request, the pair of users form an undirected social tie, called Friends. A web of Friends defines a social network of mixi. We adopted degree as the most basic network-related independent variable. The degree is the number of neighbors (i.e., Friends), and denoted by 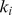 for user 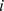. The system of mixi allows a user to own at most degree 1000. As is consistent with the previous analysis of a much smaller data set of mixi [34], the degree distributions for both groups are long tailed (Figure 2). A small degree is an indicator of social isolation.

#### Local clustering coefficient

**Figure 4:**
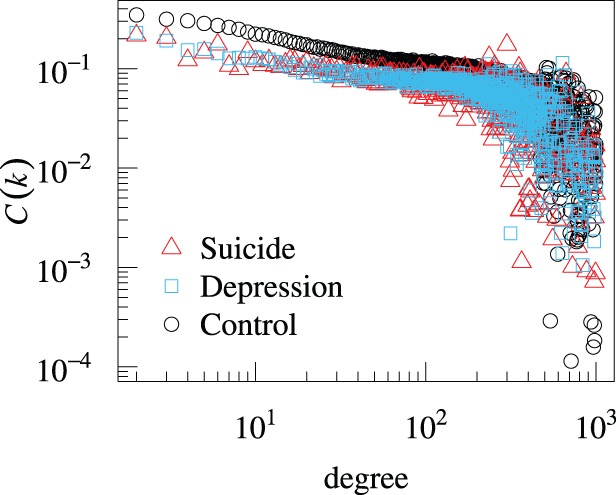
Examples of the degree (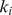) and the local clustering coefficient (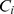). The shown values of 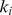 and 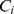 are for the nodes shown by the filled circles.

We quantified transitivity, or the density of triangles around a user, by the local clustering coefficient, denoted by 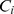 for user 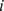. A directed-link version of the same quantity was used in the Bearman–Moody study. For user 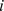 having degree 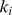, there can be maximum 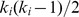 triangles that include user 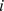. We defined 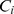 as the actual number of triangles that included 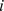 divided by 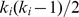. Examples are shown in Figure 4. By definition, 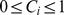. We discarded the users with 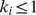 because 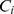 was defined only for users with 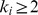. 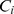 quantifies the extent to which neighbors of user 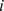 are adjacent to each other [13], [38]. If 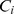 is large, the user is probably embedded in close-knit social groups [12], [13], [38]. A small 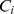 value is an indicator of social isolation. As in many networks [13], 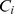 decreases with 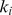 in both suicide and control groups (Figure 3). The results are consistent with those in the previous study in which the average 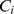 obtained without categorizing users is roughly proportional to 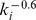 [34]. Therefore, we carefully distinguished the influence of 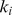 and 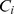 on suicide ideation by combining univariate and multivariate regressions.

#### Homophily

Suicide may be a contagious phenomenon (e.g., [6], [22]–[26]). If so, a user is inclined to suicide ideation when a neighbor in the social network is. Therefore, we adopted the fraction of neighbors with suicide ideation as an independent variable. It should be noted that, even if a user with suicide ideation has relatively many friends with suicide ideation, it does not necessarily imply that suicide is contagious. Homophily may be a cause of such assortativity. In this study, we did not attempt to distinguish the effect of imitation and homophily. The differentiation would require analysis of temporal data [20], [21]. Nevertheless, for a notational reason, we refer to the fraction of neighbors as the homophily variable.

#### Registration period

A user that registered to mixi long time ago may be more active and own more resources in mixi than new users. Such an experienced user may tend to simultaneously have, for example, a large community number, large degree, and perhaps high activities in various communities including suicidal ones. To control for this factor, we measured the registration period defined as the number of days between the registration date and January 23, 2012.

### Analysis of Depressive Symptoms

To define depression-related community, we identified the communities satisfying the five criteria as in the case of suicidal community, but with the term suicide in the community name replaced by depression (“utsu” in Japanese). There were ten such communities. We excluded three of them because their names include positive words (let’s overcome, resume one’s place in society, cure; translations by the authors). We defined the remaining seven communities, summarized in Table 9, to represent depressive symptoms of users. The depression group is the set of active users that belongs to at least one depression-related community listed in Table 9. The depression group contains 24410 users.

**Table 9:** Statistics of depression-related communities.

ID	Date of creation	No. users	No. active	Fraction of	No.	No. active
	(day/month/year)		users	active users ( 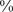 )	comments	topics
1	06/04/2004	15618	8605	54.7	14466	52
2	06/02/2006	13082	9674	72.8	1008	16
3	08/12/2004	4948	2845	56.5	782	17
4	22/04/2006	4606	2907	60.4	221	30
5	28/01/2008	3406	2321	65.0	1350	24
6	09/12/2004	3464	2039	58.2	851	20
7	21/12/2004	2440	1367	54.2	535	5

For a technical reason, we collected the number of members for communities 1, 2, 3, and 6 on November 2, 2011 and communities 4, 5 and 7 on November 4, 2011.

### Ethics Statement

Mixi approved the provision of the data.

## References

[1] Chambers A (2010) Japan: ending the culture of the ‘honourable’ suicide. The Guardian (3 August 2010).

[2] US Bureau of the Census (2012). Statistical abstract of the United States.

[3] Durkheim E (1951) Suicide. New York: Free Press.

[4] Trout DL (1980) The role of social isolation in suicide. Suicide Life-Threatening Behav 10: 10–23. 10.1111/j.1943-278x.1980.tb00693.x 7361340

[5] Joiner Jr TE, Brown JS, Wingate LR (2005) The psychology and neurobiology of suicidal behavior. Annu Rev Psychol 56: 287–314. 15709937 10.1146/annurev.psych.56.091103.070320

[6] Wray M, Colen C, Pescosolido B (2011) The sociology of suicide. Annu Rev Sociol 37: 505–528.

[7] Putnam RD (2000) Bowling Alone. New York: Simon & Schuster.

[8] Pescosolido BA, Georgianna S (1989) Durkheim, suicide, and religion: toward a network theory of suicide. Amer Sociol Rev 54: 33–48. 11616426

[9] Bearman PS (1991) The social structure of suicide. Sociol Forum 6: 501–524.

[10] Berkman LF, Glass T, Brissette I, Seeman TE (2000) From social integration to health: Durkheim in the new millennium. Soc Sci Med 51: 843–857. 10972429 10.1016/s0277-9536(00)00065-4

[11] Kawachi I, Berkman LF (2001) Social ties and mental health. J Urban Health 78: 458–467. 11564849 10.1093/jurban/78.3.458 PMC3455910

[12] Wasserman S, Faust K (1994) Social Network Analysis. New York: Cambridge University Press.

[13] Newman MEJ (2010) Networks – An introduction. Oxford: Oxford University Press.

[14] Bearman PS, Moody J (2004) Suicide and friendships among American adolescents. 10.2105/ajph.94.1.89 PMC1449832 14713704

[15] Cui S, Cheng Y, Xu Z, Chen D, Wang Y (2010) Peer relationships and suicide ideation and attempts among Chinese adolescents. Child Care Health Dev 37: 692–702. 21198776 10.1111/j.1365-2214.2010.01181.x

[16] Krackhardt D (1999) The ties that torture: Simmelian tie analysis in organizations. Research in the Sociology of Organizations 16: 183–210.

[17] Palla G, Derényi I, Farkas I, Vicsek T (2005) Uncovering the overlapping community structure of complex networks in nature and society. Nature 435: 814–818. 15944704 10.1038/nature03607

[18] Onnela JP, Saramäki J, Hyvönen J, Szabó G, Lazer D, et al (2007) Structure and tie strengths in mobile communication networks. Proc Natl Acad Sci USA 104: 7332–7336. 17456605 10.1073/pnas.0610245104 PMC1863470

[19] Backstrom L, Huttenlocher D, Kleinberg J, Lan X (2006) Group formation in large social networks: membership, growth, and evolution. Proceedings of the 12th ACM SIGKDD International Conference on Knowledge Discovery and Data Mining : 44–54.

[20] Aral S, Muchnik L, Sundararajan A (2009) Distinguishing influence-based contagion from homophily-driven diffusion in dynamic networks. Proc Natl Acad Sci USA 106: 21544–21549. 20007780 10.1073/pnas.0908800106 PMC2799846

[21] Shalizi CR, Thomas AC (2011) Homophily and contagion are generically confounded in observational social network studies. Sociol Methods Res 40: 211–239. 22523436 10.1177/0049124111404820 PMC3328971

[22] Mann JJ (2002) A current perspective of suicide and attempted suicide. Ann Intern Med 136: 302–311. 11848728 10.7326/0003-4819-136-4-200202190-00010

[23] Baller RD, Richardson KK (2002) Social integration, imitation, and the geographic patterning of suicide. Amer Soc Rev 67: 873–888.

[24] Romer D, Jamieson PE, Jamieson KH (2006) Are news reports of suicide contagious? A stringent test in six U. S. cities. J Communication 56: 253–270.

[25] Hedström P, Liu KY, Nordvik MK (2008) Interaction domains and suicide: a population-based panel study of suicides in Stockholm, 1991–1999. Soc Forces 87: 713–740.

[26] Baller RD, Richardson KK (2009) The “dark side” of the strength of weak ties: the diffusion of suicidal thoughts. J Health Soc Behav 50: 261–276. 19711805 10.1177/002214650905000302

[27] Gould MS, Wallenstein S, Davidson L (1989) Suicide clusters: a critical review. Suicide Life-Threatening Behav 19: 17–29. 10.1111/j.1943-278x.1989.tb00363.x 2652386

[28] Christakis NA, Fowler JH (2009) Connected. New York: Little, Brown and Company.

[29] Rosenquist JN, Fowler JH, Christakis NA (2011) Social network determinants of depression. Mol Psychiatry 16: 273–281. 20231839 10.1038/mp.2010.13 PMC3832791

[30] Lyons R (2011) The spread of evidence-poor medicine via flawed social-network analysis. Stat Politics Policy 2: Article 2.

[31] VanderWeele TJ (2011) Sensitivity analysis for contagion effects in social networks. Sociol Methods Res 40: 240–255. 25580037 10.1177/0049124111404821 PMC4288024

[32] Brezo J, Paris J, Turecki G (2006) Personality traits as correlates of suicidal ideation, suicide attempts, and suicide completions: a systematic review. Acta Psychiatr Scand 113: 180–206. 16466403 10.1111/j.1600-0447.2005.00702.x

[33] Martin D (2010) What Americans do online: social media and games dominate activity. Nielsen News, Online (2 August 2010).

[34] Yuta K, Ono N, Fujiwara Y (2007). A gap in the community-size distribution of a large-scale social networking site.

[35] Ugander J, Karrer B, Backstrom L, Marlow C (2011). The anatomy of the Facebook social graph.

[36] Stine RA (1995) Graphical interpretation of variance inflation factors. Am Stat 49: 53–56.

[37] Tufféry S (2011) Data Mining and Statistics for Decision Making (2nd edition). Chichester: Willey.

[38] Watts DJ, Strogatz SH (1998) Collective dynamics of ‘small-world’ networks. Nature 393: 440–442. 9623998 10.1038/30918

