# Correction: Suicide Ideation of Individuals in Online Social Networks

**DOI:** 10.1371/annotation/d589857d-b3c6-4a16-acfe-423f9bf529f1

**Published:** 2014-01-06

**Authors:** Naoki Masuda, Issei Kurahashi, Hiroko Onari

This article was republished on January 6th, 2014 because of the incorrect order of the tables. Please download this article again to view the tables in the correct order.

